# Comparative in vitro and in silico evaluation of the toxic effects of metformin and/or ascorbic acid, new treatment options in the treatment of Melasma

**DOI:** 10.1093/toxres/tfaf025

**Published:** 2025-02-27

**Authors:** Hülya Tezel Yalçın, Deniz Arca Çakır, Anıl Yirün, Sonia Sanajou, Gözde Işık, Özlem Bozdemir, İbrahim Özçelik, Merve Güdül Bacanlı, Naciye Dilara Zeybek, Terken Baydar, Pınar Erkekoğlu

**Affiliations:** Hacettepe University Faculty of Pharmacy, Department of Pharmaceutical Toxicology, Sıhhiye Ankara 06100, Turkey; Hacettepe University Vaccine Institute, Department of Vaccine Technology, Sıhhiye Ankara 06100, Turkey; Çukurova University Faculty of Pharmacy, Department of Pharmaceutical Toxicology, Balcalı Sarıçam 01250 Adana, Turkey; Hacettepe University Faculty of Pharmacy, Department of Pharmaceutical Toxicology, Sıhhiye Ankara 06100, Turkey; Hacettepe University Vaccine Institute, Department of Vaccine Technology, Sıhhiye Ankara 06100, Turkey; Hacettepe University Faculty of Medicine, Department of Histology and Embryology, Sıhhiye Ankara 06100, Turkey; Hacettepe University Graduate School of Health Sciences, Department of Stem Cell Sciences, Sıhhiye Ankara 06100, Turkey; Faculty of Pharmacy, Department of Toxicology, Erzincan Binali Yildirim University, Yalnızbağ Erzincan 24002, Turkey; Faculty of Pharmacy, Department of Toxicology, Health Sciences University, Keçiören, Ankara 06010, Turkey; Hacettepe University Faculty of Medicine, Department of Histology and Embryology, Sıhhiye Ankara 06100, Turkey; Hacettepe University Faculty of Pharmacy, Department of Pharmaceutical Toxicology, Sıhhiye Ankara 06100, Turkey; Hacettepe University Faculty of Pharmacy, Department of Pharmaceutical Toxicology, Sıhhiye Ankara 06100, Turkey; Hacettepe University Vaccine Institute, Department of Vaccine Technology, Sıhhiye Ankara 06100, Turkey

**Keywords:** hyperpigmentation, MNT-1 cells, In vitro, ascorbic acid, metformin, In silico

## Abstract

Melasma is a chronic condition that leads to the buildup of melanin pigment in the epidermis and dermis due to active melanocytes. Even though it is considered a non-life-threatening condition, pigment disorders have a negative impact on quality of life. Since melasma treatment is not sufficient and complicated, new treatment options are sought. Research on metformin and ascorbic acid suggested that they might be used against melasma in the scope of “drug repositioning.”The MNT-1 human melanoma cell line was used to assess the effects of metformin, ascorbic acid, and metformin+ascorbic acid combination on cytotoxicity and oxidative stress. Melanin, cAMP, L-3,4-dihydroxyphenylalanine (L-DOPA) and tyrosinase levels were determined by commercial ELISA kits and tyrosinase gene expression was analyzed with RT-qPCR. Cytopathological evaluations were performed by phase contrast microscopy. Tyrosinase expression was determined by immunofluorescence (IF) staining of MNT-1 cells. The online service TargetNet was used for biological target screening. The parameters were not significantly altered by ascorbic acid applied at non-cytotoxic concentrations. On the contrary, metformin dramatically raised tyrosinase and intracellular ROS levels. Moreover, intracellular ROS levels and tyrosinase levels were found to be considerably elevated with the combined treatment. Also, potential metformin and ascorbic acid interactions were determined. According to the results, it can be said that these parameters were not significantly altered by ascorbic acid. On the contrary, metformin dramatically raised tyrosinase and intracellular oxidative stress levels. Moreover, intracellular oxidative stress and tyrosinase levels were elevated with the combined treatment. In conclusion, individual treatments of ascorbic acid or metformin may only provide a limited effect when treating melasma and extensive in vitro and in vivo research are required.

## Introduction

Human skin protects the body against a wide variety of physical, chemical and biological agents and mechanical injury.^1–3^ It uses a special and complex mechanism called “pigmentation,” which includes important cellular components such as melanocytes in the epidermis, keratinocytes and fibroblasts in the dermis to protect itself from sunlight.^4^^,^^5^ Melanin is the pigment responsible for the pigmentation of the skin.^2^^,^^3^ Its molecular structure enables this pigment to absorb UV and visible light, thereby protecting the skin and eyes against UV radiation (UVR).^2^^,^^6^

The biosynthesis pathway of the melanin, known as the Raper Mason pathway, involves three main enzymes: tyrosinase (tyrosine oxidase, TYR), tyrosinase-related protein 1 [TRP-1 or glycoprotein 75 (gp75)] and tyrosinase-related protein 2 (TRP-2, dopachrome tautomerase or DCT).^7–9^ The oxidation of the amino acid tyrosine to 3,4-dihydroxy-phenylalanine (L-DOPA) by tyrosinase is the starting point of the biosynthesis of melanin pigments. It’s the rate-limiting step in melanin biosynthesis, because inhibition of this reaction leads to the cessation of melanin production.^10^

UVR and other factors stimulate keratinocytes to produce growth factors for melanin production. Besides UVR, paracrine, autocrine and hormonal factors regulate melanogenesis through multiple signaling pathways, including cAMP and microphthalmia-associated transcription factor (MITF).^11–15^ The transcription of melanin-producing enzymes including TYR, TRP-1, and TRP-2 as well as the regulation of other genes involved in melanosome maturation, trafficking, and distribution to keratinocytes are all regulated by MITF.^16^

Melasma is a chronic, acquired disease that results in the accumulation of brown or gray brown melanin pigment with irregular patterns in the epidermis layer and/or dermis layer, particularly in areas where the body is more exposed to sunlight.^17^ Treatment of melasma is difficult and complex due to its multifactorial etiology.^18^ Commonly used treatment methods include depigmenting agents, peeling agents, laser and beam treatments.^18^^,^^19^ However, each treatment option has different success rates and treatment-related risks.^18^^,^^20^ Among these treatment options, a retinoid derivative with hydroquinone (HQ) preparations offers the most effective treatment option.^18^^,^^21^ Several side effects have reported due to the use of HQ derivates including post-inflammatory hyperpigmentation, exogenous ochronosis, irritation, and erythema.^20^^,^^22–24^ Also, HQ is converted to p-benzoquinone metabolite due to the increase in myeloperoxidase activity when it reaches the bone marrow and it poses a potential carcinogenic risk.^25^^,^^26^

The need for novel treatment options has gained attention due to the adverse effects associated with HQ. Many studies on the use of medications with low toxicity and high efficacy in the treatment of melasma have been conducted recently. Certain medications, such as ascorbic acid and metformin, which are already in use, can be utilized in the scope of “drug repositioning.”

Metformin may inhibit the expression of genes involved in melanin synthesis such as MITF, tyrosinase, TRP-1 and TRP-2 by inhibiting cyclic AMP (cAMP) accumulation and cAMP-mediated protein phosphorylation. Thus, it may reduce the amount of melanin in melanocytes.^18^^,^^21^ L-ascorbic acid, the active form of ascorbic acid, inhibits tyrosinase activity by interacting with the copper ion in the structure of the tyrosinase enzyme.^27^ At the same time, it has a protective effect against both UV-A and UV-B radiation by reducing free radical production, which is known to increase melanogenesis.^28^

Considering all the available data, the current study was conducted to investigate the effects of metformin and/or L-ascorbic acid on cytotoxicity, oxidative stress; melanin, cAMP and L-3,4-dihydroxyphenylalanine (L-DOPA) and tyrosinase levels and tyrosinase gene expression in melanoma cell line (MNT-1). In addition to in vitro studies, metformin and L-ascorbic acid’s possible interactions in the biological environment were determined using the online biological target screening tool TargetNet.

## Materials and methods

### Chemicals, reagents, kits and apparatus

3–(4,5-dimethylthiazol-2-yl)-2,5-diphenyl tetrazolium bromide (MTT), intracellular ROS assay kit, bovine serum albumine (BSA) and Triton-X100 were purchased from Sigma-Aldrich (Mannheim, Germany). Dulbecco’s phosphate buffer saline (DPBS), Dulbecco’s modified Eagle’s Medium (DMEM), fetal bovine serum (FBS), and penicillin G/streptomycin, L-glutamine and tyrpsin EDTA were purchased from Biowest (Riverside, MO). Phosphate buffered saline (PBS) was purchased from HiMedia Laboratories LLC (Kelton, PN) Goat Anti-Mouse IgG H&L (FITC) was purchased from Abcam (Boston, MA). Cyclic adenosine monophosphate (cAMP) ELISA kit and human tyrosinase ELISA kit were purchased from Elabscience Biotechnology Inc. (Houston, TX). Human melanin ELISA kit and human L-DOPA ELISA kit were purchased from Shanghai Sunred Biological Technology Co., Ltd (Shanghai, China). Anti-tyrosinase antibody T311 was purchased from Santa Cruz Biotechnology, Inc. (Heidelberg, Germany). Dimethyl sulfoxide (DMSO) was purchased from Duchefa Biochemie B.V (Haarlem, The Netherlands). Hybrid-R and Riboex were purchased from GeneAll Biotechnology Co., Ltd, (Seoul, South Korea). Normal Goat Serum Block was purchased from Biolegend Inc. (San Diego, CA). WizScript™ cDNA Synthesis Kit (high capacity) and WizPure™ qPCR Master (SYBR) were purchased from Wizbio Solutions (Gyeonggi-do, South Korea). Metformin hydrochloride was purchased from Cayman Chemical Company (Ann Arbor, Michigan, U.S.A.). L-ascorbic acid was purchased from Doga Ilac Hammaddeleri (Istanbul, Turkey) MNT-1 cells (ATCC® CRL-3450™) were obtained from American Type Culture Collection (ATCC) (Rockville, MA). All other chemicals were from Sigma-Aldrich (Mannheim, Germany).

Throughout the experiments, a spectrophotometer/spectrofluorometer SpectraMax M2 was used (Molecular Devices, San Jose, California, U.S.A.). Cytopathological evaluations were performed by phase contrast microscope (The Olympus IX73, Tokyo, Japan).

### Cell culture and media

MNT-1 cells (ATCC® CRL-3450™) were cultured in DMEM high glucose (supplemented with 20% FBS, 0.1 mM penicillin G/streptomycin and 0.1 mM nonessential amino acids and were kept at 37 °C in an incubator with 5% CO_2_. Cultured MNT-1 cells were used in following experiments.

### Experimental groups

The experimental groups were:

Control group (C): MNT-1 cells exposed to DMEM high glucose and 0.1 mM nonessential amino acids for 24 h.

Metformin group (Met): MNT-1 cells exposed to 50 μM metformin according to the results obtained from cytotoxicity experiments in DMEM high glucose and 0.1 mM nonessential amino acids for 24 h.

Ascorbic acid group (Asc): MNT-1 cells exposed to 0.5 μM ascorbic acid according to the results obtained from cytotoxicity experiments in DMEM high glucose and 0.1 mM nonessential amino acids for 24 h.

Metformin+Ascorbic acid group (Asc + Met): MNT-1 cells were exposed to 50 μM metformin and 0.5 μM ascorbic acid according to the results obtained from cytotoxicity experiments in DMEM high glucose and 0.1 mM nonessential amino acids for 24 h.

### Ell viability

MTT assay was performed to determine the cytotoxic effects of metformin and ascorbic acid.^29^ The MNT-1 cells were incubated with 50 μM, 100 μM, 250 μM, 500 μM, 1,000 μM, 2,500 μM and 5,000 μM metformin and 0.1 μM, 0.5 μM, 1 μM, 5 μM, 10 μM, 20 μM, 25 μM ascorbic acid for 24 hours. After 24 h incubation, the yellow tetrazolium compound MTT (0,5 mg/mL) solution was applied. MTT transforms into purple formazan crystals as a result of an increase in dehydrogenase activity in the mitochondria of proliferating cells. Formazan crystals were dissolved by adding DMSO and cell viability was measured by determining absorbance values in a spectrophotometer at 570 nm.^29^^,^^30^ The cell viability was considered as 100% for control group and the cell viability of other study groups was given as % of control. The highest doses that did not reduce cell viability were selected for both metformin and L-ascorbic acid.

### Determination of intracellular reactive oxygen species (ROS)

ROS was determined fluorometrically by using 5- and 6-chloromethyl-2′,7′-dichlorodihydrofluorescein diacetate (CMH_2_DCFDA), which is a non-fluorescent probe. CMH_2_DCFDA is converted to 2′,7′ dichlorofluorescein (CM-DCF) by metabolically active cells. By measuring fluorescence (λ_ex_ = 540 and λ_em_ = 570 nm) formed by this conversion, the levels of ROS was determined. The amount of intracellular ROS produced by the control cells was assumed as 100%, and the amount of ROS produced by the other study groups was calculated as % of the control.

### Cytopathology

Cytopathological evaluations of the treatment groups were done under phase contrast microscope (The Olympus IX73, Tokyo, Japan).

### Immunofluorescence microscopy

Tyrosinase expression is determined by immunofluorescence (IF) staining of MNT-1 cells. In this study, indirect immunofluorescence (IF) staining was used for tyrosinase enzyme. Tyrosinase antibody (T311) was used as the primary antibody. T311 is a mouse monoclonal IgG2aκ tyrosinase antibody developed against recombinant tyrosinase of human origin. The secondary antibody is conjugated with fluorescein isothiocyanate (FITC) for IF staining.

Control MNT-1 cells, negative control MNT-1 cells and MNT-1 cells treated with 50 μM metformin hydrochloride, 0.5 μM ascorbic acid and 50 μM metformin hyrdochloride+0.5 μM ascorbic acid, respectively were seeded into 8-well chamber slides. After the 24 h incubation, the cells were rinsed briefly in PBS, fixed at room temperature for 15 min with % 4 paraformaldehyde and permeabilized with 0.1% Triton X-100 in PBS. After permeabilization, cells were treated with blocking solution with goat serum for 30 minutes at room temperature. After the blocking serum was removed, T311 (sc-20,035) diluted 1:200 with 2.5% BSA-PBS was added to the wells. This procedure was performed for all groups except the negative control group. Cells with primary antibody (T311) added were incubated at +4 °C for one night.

After the incubation, cells were washed 3 times with PBS for 5 min. The seconder antibody Goat Anti-Mouse IgG H&L (FITC) (ab6785) diluted 1:500 with 1% normal human serum (Sigma Aldrich, Mannheim, Germany) was added to the cells and kept in the dark at room temperature for 1 hour. At the end of the 1 h, cells were washed 3 times for 5 min with PBS. 10 μM of 4′,6-diamidino-2-phenylindole (DAPI) was added to the cells to stain the nuclei of the cells. Then the fluorescence of cells in each group was visualized using Leica DM6000B fluorescent-attached research microscope (Wetzlar, Germany) and Leica DC490 digital camera (Wetzlar,Germany).

### Immunfluorescence intensity measurement

In the experimental groups, immunofluorescence intensity measurements were made using Image Processing and Analysis in Java [Image J, US National Institutes of Health, Bethesda, MA and Laboratory for Optical and Computational Instrumentation (LOCI), University of Wisconsin, Madison, WI] analysis program. For this purpose, 40 cells were marked in 5 fields at X40 magnification and “corrected total cell fluorescence (CTCF)” calculation for each group was made using the formula “CTCF= Int Den - (Average of background intensity X Cell area)”. The average for each group was calculated and analyzed by drawing graphics in the GraphPad prism program (Boston, MA).

### Determination of tyrosinase levels

Tyrosinase levels were determined by an ELISA kit using the sandwich-ELISA principle. Optical density was measured spectrophotometrically at a wavelength of 450 nm. By comparing the optical density of the samples with the standard curve, the concentration of human TYR in the samples was calculated.

### Determination of tyrosinase gene expression

mRNA was isolated from MNT-1 cells with RiboEx™ and Hybrid-R™ according to the manufacturer’s instructions (GeneAll®). After mRNA isolation, WizScript™ (Wizbio) cDNA Synthesis Kit (High Capacity) was used for cDNA synthesis. Reaction conditions for cDNA synthesis are shown in Table 1.

**Table 1:** cDNA synthesis reaction conditions and real-time qPCR reaction conditions.

**DNA Synthesis Reaction Conditions**	**Step 1**	**Step 2**	**Step 3**	**Step 4**
**Temperature (°C)**	25	37	85	4
**Time**	10 min	120 min	5 min	-
**PCR Steps and Reaction Conditions**	**Temperature (°C)**	**Time**	**Cycle**	
**Initial Denaturation**	95	300 sec	1	
**Denature**	95	15 sec	40	
**Anneal**	55–68	60 sec		

WizPure™ qPCR Master (SYBR) (Wizbio) kit was used for RT-qPCR. The designed primer sequences used for PCR were as follows: tyrosinase, TCTTCTTGTTGCGGTGGGAA (forward), TGATGCTGGGCTGAGTAAGT (reverse); β-actin, CATCCTCACCCTGAAGTACC (forward), TGAAGGTCTCAAACATGATCTG (reverse). Real-Time qPCR reaction was performed on Applied Biosystems™ QuantStudio™ 5 Real-Time PCR System (Waltham, MA). The reaction conditions used for RT-qPCR are given in Table 1.

### Calculation of relative quantitation

Quantification of mRNA expressions was normalized to the control group using the ACTB transcript as a reference. “∆∆Ct Method” was used in the calculation of relative quantification.

### Determination of melanin levels

A human melanin ELISA kit using the sandwich ELISA principle was used to determine melanin content. The amount of melanin in the standard solutions and samples was measured spectrophotometrically at a wavelength of 450 nm.

### Determination of cAMP levels

cAMP levels were determined by an ELISA kit using the competitive-ELISA principle. Optical density was measured spectrophotometrically at a wavelength of 450 nm.

### Determination of L-DOPA levels

A L-DOPA ELISA kit using the sandwich ELISA principle was used to determine the amount of L-DOPA. The amount of L-DOPA in the standard solutions and samples was measured spectrophotometrically at a wavelength of 450 nm.

### Determination of protein levels

A modification of the Lowry method was used for protein measurement. The method is based on the reactivity of peptide nitrogens with copper [II] ions under alkaline conditions and the subsequent reduction of foline phenol reagent. First, 40 μL deionized water, albumin standard solutions, and samples were transferred to the ELISA plate wells. Copper reagent (freshly prepared before the experiment, 40 μL) was added to the wells. The wells were incubated at room temperature for 10 min. Foline phenol solution (120 μL) was added to the wells. The plate was incubated in a 50 °C water bath for 10 min. The absorbance values were read at 540 nm. The protein levels in the cells were calculated from the standard curve obtained from the standards.

### TargetNet for in silico approaches

In order to investigate the effects of metformin and L-ascorbic acid on melasma, their possible interactions in the biological environment were determined using the biological target screening method. Then, the effects of the identified targets on melanin synthesis were investigated. The online service TargetNet was used for biological target screening.^31^ TargetNet uses quantitative structure–activity relationship (QSAR) models built with data from BindingDB to examine the activity of the molecule on 623 different human proteins. In this study, extended connectivity fingerprint type P4 (ECFP4) was used to identify the molecule in TargetNet system. In addition, to improve the accuracy of the results, models with AUC value of 0.7 or higher, which is a measure of accuracy in the classifications of QSAR model, were used. All models prepared for the targets can be downloaded from the protein information page on TargetNet, and the accuracy scores for different molecular fingerprint types that can be selected on the molecule entry screen can also be accessed from this page.

### Statistical analysis

Statistical Package for Social Sciences Program (SPSS) 17.0 (Chicago, IL) was used for the statistical analysis. Comparison of the results obtained from all groups was made with Kruskal–Wallis one-way analysis of variance, followed by Student’s t test. Results were given as mean ± standard deviation (SD). p value <0.05 was considered statistically significant. The mean corrected total cell fluorescence (CTCF) was determined for each group with the Image J program. Data were given as mean ± standard error of mean (SEM), p value <0.0001 was considered statistically significant.

## Results

### Cell viability

**Fig. 1:**
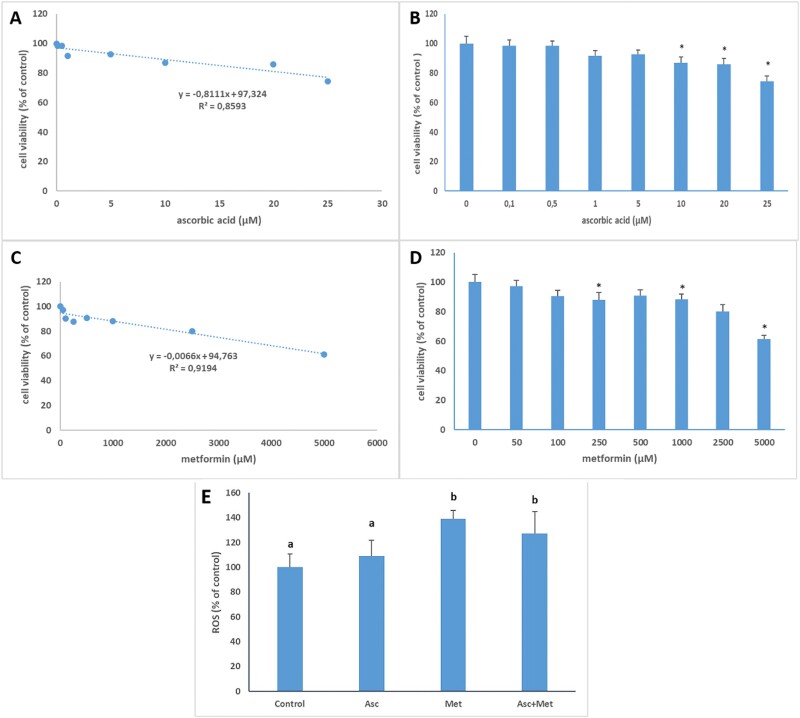
A and B: Cell viability after ascorbic acid exposure in MNT-1 cells. C and 1D: Cell viability after metformin exposure in MNT-1 cells. E: ROS levels in study groups.

The cell viability after ascorbic acid exposure in MNT-1 cells is given in Fig. 1A and 1B. The cell viability after metformin exposure in MNT-1 cells is shown in Fig. 1C and 1D. The cell viability decreased dose-dependently after both ascorbic acid and metformin exposures. Cell viability was lower than control cells in ascorbic acid-applied cells after 10, 20 and 25 μM applications. and in metformin-exposed cells after 250, 1,000 and 5,000 μM applications. Reducing cell proliferation is not a practical treatment strategy for melasma since hyperfunctioning melanocytes contribute to hyperpigmented skin.^32^ Thus, for both metformin and L-ascorbic acid, the highest doses (50 μM for metformin and 0.5 μM for L-ascorbic acid) that did not impair cell viability were chosen.

### Intracellular ROS levels

ROS levels in study groups are shown in Fig. 1E. ROS levels were insignificantly higher in ascorbic acid treated cells (9%) while in both metformin (39%) and ascorbic acid+metformin treated group (27%) ROS levels were markedly higher than control in all exposed groups (*P* < 0.05).

### Cytopathology

**Fig. 2:**
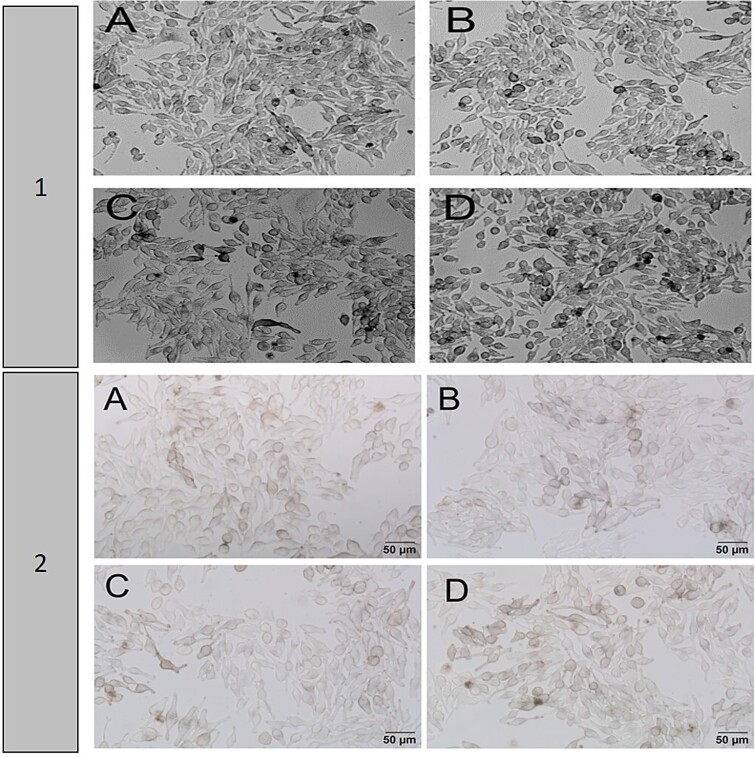
Phase contrast microscopy images of the experimental groups. 1st panel: A: Control, B: Ascorbic acid (Asc), C: Metformin (met), D: Combined application (Asc + met) (phase contrast X100), 2nd panel: A: Control, B: Ascorbic acid (Asc), C: Metformin (met), D: Combined application (Asc + met) (phase contrast X400).

Cytopathological evaluations are shown in Fig. 2. The fibroblast-like spindle-shaped MNT-1 cells attached to the flask base were observed in the samples belonging to the experimental groups under a phase contrast microscope. Experimental groups contained a similar number of cells. It was observed that MNT-1 cells had euchromatic nuclei, and dark granules were present in the cytoplasm surrounding the nucleus. The cytoplasm appeared brown and black pigmented due to the presence of these granules. In the control group, MNT-1 cells with brown pigmentation in the cytoplasm were predominant. The brown pigmentation within MNT-1 cells was lighter in the ascorbic acid and metformin applied groups compared to the control. Cells with large non-dividing cytoplasm were observed in the metformin group. The brown pigmentation was also observed in the ascorbic acid+metformin group and MNT-1 cells were observed at a similar density compared to the control group.

At a higher magnification of the experimental groups (Fig. 2-Panel 2), we observed brown granules (melanin) in the cytoplasm, and pigmentation was observed around the nucleus. MNT-1 cells with brown pigmentation were observed less frequently in the groups treated with ascorbic acid and metformin compared to the control group, while in the combined group the pigmentation was with a similar density to the control group.

### Immunofluorescence microscopy

**Fig. 3:**
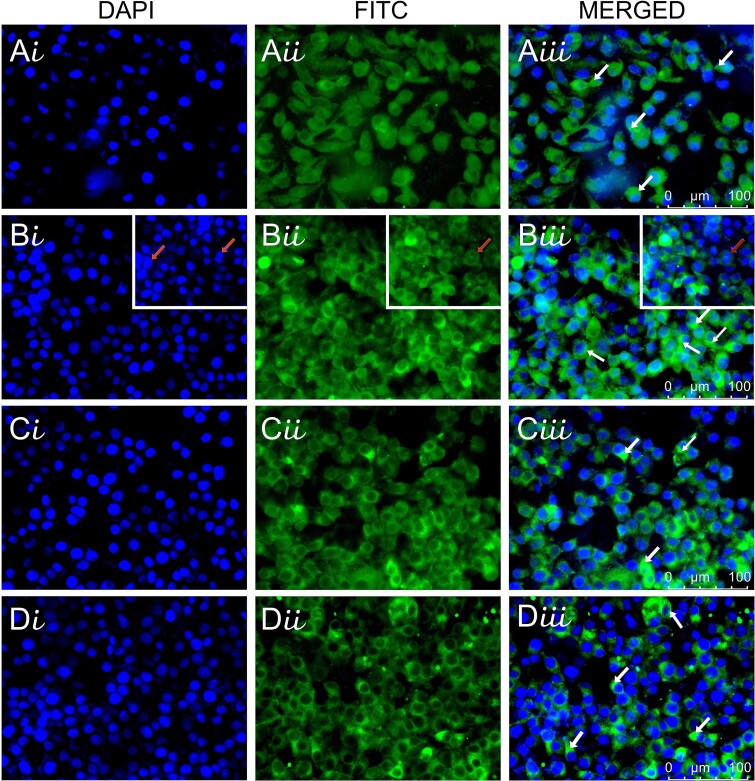
Indirect immunofluorescence labeling with anti-tyrosinase in experimental groups. In the labeling with anti-tyrosinase, positive immunoreactivity with anti-tyrosinase (arrow) is observed in the cytoplasm of the cells. Mitotic cells (arrow in inset B) were observed in the ascorbic acid group. A: Control, B: Ascorbic acid (Asc), C: Metformin (met), D: Combined application (Asc + met). i: DAPI, ii: FITC, iii: Merged, X400.

MNT-1 cells in the experimental groups exhibited a granular, punctate anti-tyrosinase immunofluorescence labeling in the cytoplasm (Fig. 3). A number of mitotic cells were observed in the examination of the ascorbic acid group.

**Fig. 4:**
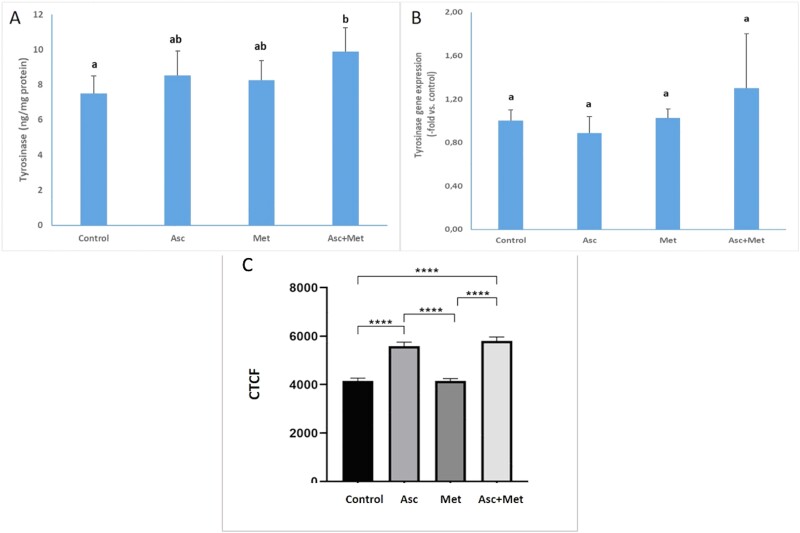
A Tyrosinase levels. A,bBars that do not share the same letters are significantly different from each other (*P* < 0.05). B: Tyrosinase gene expression. A,bBars that do not share the same letters are significantly different from each other (*P* < 0.05). C: Quantitative fluorescent image analysis of indirect immunofluorescent labeling with anti-tyrosinase in the experimental groups. Mean CTCF calculated from images at 40X magnification of experimental groups after anti-tyrosinase immunofluorescence labeling (CTCF in 5 fields for each group and at least 40 cells in each field). Data are given as mean ± standard error of mean (SEM). ^*^^*^^*^^*^P < 0.0001.

For the intensity measurements, the mean CTCF was determined for each group by the Image J program (Fig. 4C). The mean of CTCF in the Asc and Asc + Met groups was higher than the control and this increase was statistically significant (*P* < 0.0001). In addition, when the Asc and Asc + Met groups were compared with the Met group, CTCF significantly increased in the Asc and Asc + Met groups compared to the Met group (*P* < 0.0001).

### Tyrosinase levels and tyrosinase gene expression

Tyrosinase levels and tyrosinase gene expression is given in Fig. 4A and B. Tyrosinase levels increased by 14% with ascorbic acid application, 10% with metformin application, and by 32% with combined application compared to control. A significant difference was found between the combined application and the control group in terms of tyrosinase levels (*P* < 0.05). Ascorbic acid decreased tyrosinase gene expression by 11%, while metformin increased it by 3%. Tyrosinase gene expression increased by 30% with combined administration.

### Melanin, cAMP and L-DOPA levels

Melanin, cAMP and L-DOPA levels are given in Table 2. With the application of ascorbic acid, melanin levels decreased by 12% compared to the control. With metformin application, melanin levels decreased by 22% compared to control, and melanin levels decreased by 9% with combined application. However, no significant difference was found between the groups in terms of melanin levels (*P* > 0.05, all).

**Table 2:** Melanin, cAMP and L-DOPA levels in the study groups.

	Melanin (ng/mg protein)	cAMP (ng/mg protein)	L-DOPA (ng/mg protein)
**Control**	27.92 ± 7.77^a^	3.64 ± 0.61^a^	19.99 ± 3.22^ab^
**Asc**	24.57 ± 7.72^a^	3.09 ± 0.66^a^	25.35 ± 0.58^a^
**Met**	21.67 ± 3.84^a^	3.43 ± 0.86^a^	19.88 ± 1.17^b^
**Asc + Met**	25.34 ± 4.72^a^	3.25 ± 0.80^a^	23.59 ± 3.97^ab^

^a,b^Columns that do not share the same letters are significantly different from each other (p < 0.05).

Ascorbic acid decreased cAMP levels by 15% compared to the control. With metformin administration, cAMP levels decreased by 6% compared to control. cAMP levels decreased by 11% with combined administration vs. control group. However, there was no significant difference between the groups in terms of cAMP levels (*P* > 0.05).

L-DOPA levels increased by 27% with ascorbic acid application compared to control. However, due to the high standard of variation within the ascorbic acid group, this change was not statistically significant. L-DOPA levels increased by 18% with combined administration compared to control (*P* > 0.05). A significant difference was determined between the administration of ascorbic acid and the administration of metformin in terms of L-DOPA levels (*P* < 0.05).

### In silico approaches

The results of the study obtained from TargetNet are given as the interaction probability scores of all biological targets. In TargetNet, these values are expressed as a value between 0 and 1. In this study, targets with a probability of 0.7 and above are accepted as “interacting.” Potential metformin interactions predicted by TargetNet is given in Table 3 and possible L-ascorbic acid interactions predicted by TargetNet can be found in Table 4.

**Table 3:** Potential metformin interactions predicted by TargetNet.

TargetNet Protein Information Page	TargetNet Protein Code	Name of Protein	Estimated Interaction Probability
http://targetnet.scbdd.com/home/details/P07478	P07478	Trypsin-2	1.0
http://targetnet.scbdd.com/home/details/P29476	P29476	Nitric oxide synthase, brain	1.0
http://targetnet.scbdd.com/home/details/P29477	P29477	Nitric oxide synthase, inducible^a^	1.0
http://targetnet.scbdd.com/home/details/P29474	P29474	Nitric oxide synthase, endothelial^a^	1.0
http://targetnet.scbdd.com/home/details/P29475	P29475	Nitric oxide synthase, brain	1.0
http://targetnet.scbdd.com/home/details/P19634	P19634	Sodium/hydrogen exchanger 1	1.0
http://targetnet.scbdd.com/home/details/P35228	P35228	Nitric oxide synthase, inducible^a^	1.0
http://targetnet.scbdd.com/home/details/O42713	O42713	Polyphenol oxidase 2	1.0
http://targetnet.scbdd.com/home/details/P00749	P00749	Urokinase-type plasminogen activator	1.0
http://targetnet.scbdd.com/home/details/P08173	P08173	Muscarinic acetylcholine receptor M4^a^	1.0
http://targetnet.scbdd.com/home/details/P22086	P22086	Alpha-2C adrenergic receptor^a^	1.0
http://targetnet.scbdd.com/home/details/P43681	P43681	Neuronal acetylcholine receptor subunit alpha-4	1.0
http://targetnet.scbdd.com/home/details/P22748	P22748	Carbonic anhydrase 4	1.0
http://targetnet.scbdd.com/home/details/P23280	P23280	Carbonic anhydrase 6	1.0
http://targetnet.scbdd.com/home/details/P28566	P28566	5-hydroxytryptamine receptor 1E	0.999
http://targetnet.scbdd.com/home/details/P47898	P47898	5-hydroxytryptamine receptor 5A	0.999
http://targetnet.scbdd.com/home/details/P43166	P43166	Carbonic anhydrase 7	0.999
http://targetnet.scbdd.com/home/details/Q9Y2D0	Q9Y2D0	Carbonic anhydrase 5B, mitochondrial	0.999
http://targetnet.scbdd.com/home/details/P00760	P00760	Cationic trypsin	0.998
http://targetnet.scbdd.com/home/details/Q96EB6	Q96EB6	NAD-dependent protein deacetylase sirtuin-1^a^	0.998
http://targetnet.scbdd.com/home/details/O43570	O43570	Carbonic anhydrase 12	0.998
http://targetnet.scbdd.com/home/details/P14324	P14324	Farnesyl pyrophosphate synthase	0.998
http://targetnet.scbdd.com/home/details/P12527	P12527	Arachidonate 5-lipoxygenase	0.998
http://targetnet.scbdd.com/home/details/Q8IXJ6	Q8IXJ6	NAD-dependent protein deacetylase sirtuin-2^a^	0.997
http://targetnet.scbdd.com/home/details/P21396	P21396	Amine oxidase [flavin-containing] A	0.996
http://targetnet.scbdd.com/home/details/O95136	O95136	Sphingosine 1-phosphate receptor 2	0.995
http://targetnet.scbdd.com/home/details/P07477	P07477	Trypsin-1	0.994
http://targetnet.scbdd.com/home/details/P10980	P10980	Muscarinic acetylcholine receptor M2^a^	0.991
http://targetnet.scbdd.com/home/details/Q04206	Q04206	Transcription factor p65	0.99
http://targetnet.scbdd.com/home/details/P16050	P16050	Arachidonate 15-lipoxygenase	0.985
http://targetnet.scbdd.com/home/details/Q99720	Q99720	Sigma non-opioid intracellular receptor 1	0.985
http://targetnet.scbdd.com/home/details/P00750	P00750	Tissue-type plasminogen activator	0.979
http://targetnet.scbdd.com/home/details/P35869	P35869	Aryl hydrocarbon receptor	0.967
http://targetnet.scbdd.com/home/details/O00748	O00748	Cocaine esterase	0.965
http://targetnet.scbdd.com/home/details/P68400	P68400	Casein kinase II subunit alpha	0.959
http://targetnet.scbdd.com/home/details/P55211	P55211	Caspase-9^a^	0.957
http://targetnet.scbdd.com/home/details/P09483	P09483	Neuronal acetylcholine receptor subunit alpha-4	0.951
http://targetnet.scbdd.com/home/details/P18089	P18089	Alpha-2B adrenergic receptor^a^	0.913
http://targetnet.scbdd.com/home/details/P08482	P08482	Muscarinic acetylcholine receptor M1^a^	0.911
http://targetnet.scbdd.com/home/details/P31941	P31941	DNA dC- > dU-editing enzyme APOBEC-3A	0.883
http://targetnet.scbdd.com/home/details/O60755	O60755	Galanin receptor type 3	0.879
http://targetnet.scbdd.com/home/details/P23443	P23443	Ribosomal protein S6 kinase beta-1	0.875
http://targetnet.scbdd.com/home/details/Q14833	Q14833	Metabotropic glutamate receptor 4	0.867
http://targetnet.scbdd.com/home/details/P15917	P15917	Lethal factor	0.859
http://targetnet.scbdd.com/home/details/P51812	P51812	Ribosomal protein S6 kinase alpha-3	0.857
http://targetnet.scbdd.com/home/details/P15144	P15144	Aminopeptidase N	0.845
http://targetnet.scbdd.com/home/details/P56524	P56524	Histone deacetylase 4	0.842
http://targetnet.scbdd.com/home/details/P25025	P25025	C-X-C chemokine receptor type 2	0.826
http://targetnet.scbdd.com/home/details/Q9ULX7	Q9ULX7	Carbonic anhydrase 14	0.805
http://targetnet.scbdd.com/home/details/Q99685	Q99685	Monoglyceride lipase	0.795
http://targetnet.scbdd.com/home/details/P35218	P35218	Carbonic anhydrase 5A, mitochondrial	0.781
http://targetnet.scbdd.com/home/details/Q9H3N8	Q9H3N8	Histamine H4 receptor^a^	0.74
http://targetnet.scbdd.com/home/details/Q16548	Q16548	Bcl-2-related protein A1	0.722

^a^Those marked with an asterisk will be discussed in detail in the discussion section.

**Table 4:** Potential L-ascorbic acid interactions predicted by TargetNet.

TargetNet Protein Information Page	TargetNet Protein Code	Name of Protein	Estimated Interaction Probability
http://targetnet.scbdd.com/home/details/P47936	P47936	Cannabinoid receptor 2	1.0
http://targetnet.scbdd.com/home/details/Q9QZN9	Q9QZN9	Cannabinoid receptor 2	1.0
http://targetnet.scbdd.com/home/details/O42713	O42713	Polyphenol oxidase 2	1.0
http://targetnet.scbdd.com/home/details/P16050	P16050	Arachidonate 15-lipoxygenase	1.0
http://targetnet.scbdd.com/home/details/P23141	P23141	Liver carboxylesterase 1	1.0
http://targetnet.scbdd.com/home/details/P00491	P00491	Purine nucleoside phosphorylase	1.0
http://targetnet.scbdd.com/home/details/O95136	O95136	Sphingosine 1-phosphate receptor 2^a^	1.0
http://targetnet.scbdd.com/home/details/O00748	O00748	Cocaine esterase	1.0
http://targetnet.scbdd.com/home/details/Q16548	Q16548	Bcl-2-related protein A1	1.0
http://targetnet.scbdd.com/home/details/P14324	P14324	Farnesyl pyrophosphate synthase	1.0
http://targetnet.scbdd.com/home/details/P30305	P30305	M-phase inducer phosphatase 2	1.0
http://targetnet.scbdd.com/home/details/P51452	P51452	Dual specificity protein phosphatase 3	0.999
http://targetnet.scbdd.com/home/details/Q8N1Q1	Q8N1Q1	Carbonic anhydrase 13	0.999
http://targetnet.scbdd.com/home/details/P23280	P23280	Carbonic anhydrase 6	0.999
http://targetnet.scbdd.com/home/details/Q9NR96	Q9NR96	Toll-like receptor 9	0.999
http://targetnet.scbdd.com/home/details/Q8TDS4	Q8TDS4	Hydroxycarboxylic acid receptor 2	0.996
http://targetnet.scbdd.com/home/details/P05979	P05979	Prostaglandin G/H synthase 1	0.996
http://targetnet.scbdd.com/home/details/P42262	P42262	Glutamate receptor 2^a^	0.996
http://targetnet.scbdd.com/home/details/P08482	P08482	Muscarinic acetylcholine receptor M1^a^	0.995
http://targetnet.scbdd.com/home/details/Q9Y2D0	Q9Y2D0	Carbonic anhydrase 5B, mitochondrial	0.994
http://targetnet.scbdd.com/home/details/P55211	P55211	Caspase-9^a^	0.993
http://targetnet.scbdd.com/home/details/P35439	P35439	Glutamate receptor ionotropic, NMDA 1^a^	0.993
http://targetnet.scbdd.com/home/details/P04058	P04058	Acetylcholinesterase	0.991
http://targetnet.scbdd.com/home/details/P05186	P05186	Alkaline phosphatase, tissue-nonspecific isozyme	0.991
http://targetnet.scbdd.com/home/details/P08483	P08483	Muscarinic acetylcholine receptor M3^a^	0.987
http://targetnet.scbdd.com/home/details/Q99685	Q99685	Monoglyceride lipase	0.978
http://targetnet.scbdd.com/home/details/P20309	P20309	Muscarinic acetylcholine receptor M3^a^	0.969
http://targetnet.scbdd.com/home/details/P35869	P35869	Aryl hydrocarbon receptor	0.963
http://targetnet.scbdd.com/home/details/P14174	P14174	Macrophage migration inhibitory factor	0.959
http://targetnet.scbdd.com/home/details/P31941	P31941	DNA dC- > dU-editing enzyme APOBEC-3A	0.945
http://targetnet.scbdd.com/home/details/P15144	P15144	Aminopeptidase N	0.942
http://targetnet.scbdd.com/home/details/P22748	P22748	Carbonic anhydrase 4	0.938
http://targetnet.scbdd.com/home/details/P08173	P08173	Muscarinic acetylcholine receptor M4^a^	0.926
http://targetnet.scbdd.com/home/details/P35218	P35218	Carbonic anhydrase 5A, mitochondrial	0.915
http://targetnet.scbdd.com/home/details/O43570	O43570	Carbonic anhydrase 12	0.913
http://targetnet.scbdd.com/home/details/P34972	P34972	Cannabinoid receptor 2	0.911
http://targetnet.scbdd.com/home/details/P10980	P10980	Muscarinic acetylcholine receptor M2^a^	0.903
http://targetnet.scbdd.com/home/details/P43166	P43166	Carbonic anhydrase 7	0.899
http://targetnet.scbdd.com/home/details/P22086	P22086	Alpha-2C adrenergic receptor^a^	0.894
http://targetnet.scbdd.com/home/details/Q04609	Q04609	Glutamate carboxypeptidase 2	0.879
http://targetnet.scbdd.com/home/details/P03372	P03372	Estrogen receptor^a^	0.846
http://targetnet.scbdd.com/home/details/P28566	P28566	5-hydroxytryptamine receptor 1E	0.801
http://targetnet.scbdd.com/home/details/P29477	P29477	Nitric oxide synthase, inducible	0.793
http://targetnet.scbdd.com/home/details/P35563	P35563	5-hydroxytryptamine receptor 3A	0.792
http://targetnet.scbdd.com/home/details/Q9HC97	Q9HC97	G-protein coupled receptor 35	0.788
http://targetnet.scbdd.com/home/details/P18089	P18089	Alpha-2B adrenergic receptor^a^	0.738
http://targetnet.scbdd.com/home/details/Q96EB6	Q96EB6	NAD-dependent protein deacetylase sirtuin-1^a^	0.736

^a^Those marked with an asterisk will be discussed in detail in the discussion section.

In summary, it was found that metformin interacts with muscarinic acetylcholine receptors, nitric oxide synthase, NAD-dependent protein deacetylase sirtuin 1, sirtuin 2, caspase-9, adrenergic receptors (alpha-2b (α-2b) and alpha-2c (α-2c)) and histamine H4 receptors. L-ascorbic acid interacts with sphingosine 1-phosphate receptor 2 (S1PR2), estrogen receptors and glutamate receptors.

## Discussion

This study aimed to investigate the efficacy of metformin and ascorbic acid on melanogenesis. Although there are few studies in the literature on this topic to the best of our knowledge, there is no study that shows the combined effect of ascorbic acid and metformin on MNT-1 cells. Our results are discussed in the subsections below.

### Cell viability

It’s known that hyperfunctioning melanocytes are involved in hyperpigmented skin and reducing cell proliferation is not a realistic treatment approach for melasma.^32^ Therefore, the highest doses (0.5 μM for L-Ascorbic acid and 50 μM for metformin applied for 24 h) that did not reduce cell viability were selected for both metformin and L-ascorbic acid. We can suggest that that both ascorbic acid and metformin produce a dose-dependent cytotoxicity in MNT-1 cells. In a study, Panich et al. (2011) studied with G361 melanoma cell line to determine the cytotoxic effects of ascorbic acid and they found that ascorbic acid (15–240 μM) did not show cytotoxic effects.^33^ Lee et al. (2011) applied ascorbic acid (50, 100, 200 and 500 μM) to B16F10 cells and stated that ascorbic acid did not cause any cytotoxicity at these concentrations. Thus, at 50 and 100 μM concentrations, ascorbic acid significantly increased the cell viability.^34^ Choi et al. (2010) also applied ascorbic acid to B16F10 cells (0, 6.25, 12.5 and 25 μg/mL) and determined that ascorbic acid did not show cytotoxic effect at any of the applied concentrations.^35^ On the other hand, Tomic et al. (2011) conducted a study to investigate the effect of metformin (1–10 mM) on A375, WM9, SKMel28 and G361 melanoma cell lines and normal human melanocytes (NHM). A dose-dependent reduction in the number of melanoma cells was seen after cells were treated with metformin for 72 h in all cell lines but not in NHM. Therefore, it was suggested that normal melanocytes were found to be resistant to metformin therapy.^36^ We can conclude that cell type, applied doses and exposure time may have made a difference.

### Intracellular ROS levels

Recent studies showed that ROS are crucial for the regulation of melanogenesis. Melanogenesis-related proteins, such as tyrosinase, cAMP-responsive element binding protein and MITF are activated by the production of ROS.^37^

In the current work, we observed that metformin and combined treatment caused increases in intracellular ROS levels while ascorbic acid did not affect ROS generation. Panich et al. (2011) observed that the UVA (8 J/cm^2^) induced ROS generation in G361 melanoma cell line was decreased after ascorbic acid application at 60 and 120 μM in a dose-dependent manner.^33^ Choi et al. (2010) compared the effect of ascorbic acid on ROS formation with the effect of a multivitamin mixture (10,000 IU vitamin A, 1,000 IU vitamin D, 5 IU vitamin E, 50 mg vitamin B1, 12.7 mg vitamin B2, 15 mg vitamin B6, 500 mg ascorbic acid, 100 mg nicotinamide and 25 mg vitamin B5) using the B16F10 murine melanoma cell line. The antioxidant effect of ascorbic acid was significantly higher than the multivitamin mixture at 12.5 μg/mL and no significant difference was observed in the antioxidant effect between the two groups at 6.5 μg/mL and 25 μg/mL.^35^ On the other hand, metformin is suggested to increase the expression of apoptotic proteins, as well as ROS formation in C32 melanoma cells.^38^ Nishida et al. (2021) showed that metformin elevates mitochondrial ROS formation via nuclear factor erythroid 2–related factor 2 (Nrf2) pathway in BALB/c and C57BL/6 (B6) mice.^39^ Our results also support the results of these studies.

### Cytopathology

In a study conducted by Choi et al. (2010), no significant change in morphology was observed after ascorbic acid treatment (0, 6.25, 12.5 and 25 μg/mL) in B16F0 murine melanoma cells compared to the control.^34^ In our study, in control group, brown pigmentation was observed in the cytoplasm of MNT-1 cells. In the ascorbic acid treated group, brown pigmentation was observed lower compared to the control group. This may be due to the melanin decrease caused by ascorbic acid.

### Tyrosinase

To best of our knowledge, there is no study in the literature that evaluated the combined effect of ascorbic acid and metformin on melanoma cells using immunofluorescence labeling of tyrosinase. Immunofluorescence analysis showed a significant number of mitotic cells in the ascorbic acid-treated group. In the experimental groups, granular, punctate structures were seen in the cytoplasm after indirect immunofluorescence labeling with anti-tyrosinase. After immunofluorescence imaging, immunofluorescence intensity measurements showed that the mean CTCF calculated from images of ascorbic acid and ascorbic acid+metformin groups significantly higher than control.

Panich et al. (2011) used the L-DOPA oxidation rate to determine the cellular tyrosinase activity in the G361 melanoma cell line exposed to UVA rays. Ascorbic acid could not reduce the cellular tyrosinase activity induced by UVA.^33^ As part of this study, 0.5 μM concentration of ascorbic acid was applied to MNT-1 cells. Tyrosinase activity was not induced by UVA or any other external factor. However, ascorbic acid increased the tyrosinase levels by 13.93% compared to the control group. In a study by Lehraiki et al. (2014), researchers showed that metformin significantly decreased the number of melanin-positive cells.^40^ In this study, we observed that MNT-1 cells with brown pigmentation were lower in number in the metformin administered group and cells with large cytoplasm that could not divide were observed. On the other hand, in the combined group, brown pigmentation was observed in the MNT-1 cells, which were found to be of similar density to the control group.

In a study by Lehraiki et al. (2014), metformin was found to reduce basal levels of tyrosinase and inhibit the increases in tyrosinase levels caused by forskolin and α-melanocyte stimulating hormone (α-MSH).^40^^,^^41^ On the other hand, the researchers investigated the effects of metformin on melanogenesis related proteins in the same study as well. Administration of forskolin or α-MSH significantly increased MITF, tyrosinase and TRP1 protein levels and normal human melanocytes, while administration of metformin significantly decreased their expressions.^40^ In our study, we observed that metformin increased both tyrosinase levels and tyrosinase gene expression very slightly in contrast to the study by Lehraiki et al. (2014). However, both tyrosinase levels and tyrosinase gene expression increased with combined application of metformin and ascorbic acid.

Lee et al. (2011) stated that ascorbic acid caused a significant stimulation on cellular tyrosinase activity and increased the mRNA and protein levels of the enzyme in B16F10 cells.^34^ Choi et al (2010), analyzed the effects of the ascorbic acid (25 μg/mL) on tyrosinase expression in B16F10 melanoma cells. It was concluded that ascorbic acid did not decrease tyrosinase expression induced by α-MSH.^35^ In our study, in contrast to the studies by Lee et al. (2011) and Choi et al. (2010), ascorbic acid (0.5 μM) decreased tyrosinase gene expression in MNT-1 cells. It is thought that different concentrations of ascorbic acid, application time and different cell types may be effective in these inconsistent findings.

### Melanin, cAMP and L-DOPA levels

In the before mentioned study of Panich et al. (2011), the researchers showed that melanin content was significantly and dose-dependently decreased after application of ascorbic acid at doses of 60 μM and 120 μM in G361 melanoma cell line.^33^ However, in the study of Lee et al. (2011), the researchers observed that ascorbic acid (50–200 μM) increased cellular proliferation and melanin content in B16F10 melanoma cells.^34^ In this study, melanin levels decreased insignificantly after ascorbic acid treatment compared to the control, similar to the results of Panich et al. (2011). In the before mentioned study of Lehraiki et al. (2014), the researchers observed that 5 mM and 10 mM metformin significantly decreased melanin synthesis in basal, forskolin and α-MSH-stimulated conditions.^40^^,^^41^ In the present work, we observed that both melanin and combined application of metformin and ascorbic acid decreased melanin levels insignificantly compared to control.

In the study by Lee et al. (2011), the researchers observed that ascorbic acid did not suppress the increase in cAMP levels in B16F10 cells.^34^ In our study, ascorbic acid treatment decreased cAMP levels insignificantly in MNT-1 cells. Lehraiki et al. (2014) determined that cAMP accumulation decreased in a time-dependent manner after 10 mM metformin administration forskolin or α-MSH with 5 mM and the maximum effect was observed after 48 h.^40^ In our study, metformin and combined application of metformin and ascorbic acid decreased cAMP levels. The decrease in cAMP levels as a result of metformin administration supports the findings of Lehraiki et al. (2014).^40^

The oxidation of the amino acid tyrosine to L-DOPA by the enzyme tyrosinase is the starting point for the biosynthesis of eumelanin and pheomelanin pigments.^10^ Based on this information, L-DOPA levels were determined as well as tyrosinase levels within the scope of this study. To the best of our knowledge, there is no study in the literature investigating the effect of ascorbic acid and metformin on L-DOPA levels in melanoma cells. We observed that the application of ascorbic acid to MNT-1 cells increased L-DOPA levels compared to the control group while administration of metformin provided insignificant decrease in L-DOPA vs. control. A marked difference was determined between ascorbic acid treatment and metformin treatment in L-DOPA levels. After the combined application of metformin and ascorbic acid, L-DOPA levels were found to be insignificantly decreased compared to the control.

### In silico approaches

Possible interactions of metformin and L-ascorbic acid in the biological environment were determined using the online biological target-screening tool TargetNet. It was found that both metformin and L-ascorbic acid interact with muscarinic acetylcholine receptors, nitric oxide synthase, NAD-dependent protein deacetylase sirtuin 1 and sirtuin 2, caspase-9 and adrenergic receptors (α_2b_ and α_2c_).

Muscarinic acetylcholine receptors are a type of G-protein coupled receptor that are widely distributed in the central nervous system and the periphery. These receptors affect a wide variety of physiological functions such as heart rate regulation, smooth muscle contraction in the eye, intestine, and airways, and insulin secretion in pancreatic β-cells. They can also affect cell proliferation and differentiation processes in some cell types.^42–44^ Muscarinic acetylcholine receptors may play a role in the development of melasma by modulating melanocyte activity and, consequently, melanin synthesis.^45^^,^^46^ In our study, we found that both metformin and L-ascorbic acid interact with muscarinic acetylcholine receptors in silico.

Studies have shown that nitric oxide synthase (NOS) isoforms are differentially expressed in various melanocyte subsets.^47^ A study by Sasaki et al. (2000) reported that nitric oxide (NO) produced after UV radiation stimulates melanocytes and contributes to melanogenesis.^48^ NO is also one of the melanogenesis-stimulating factors released from cells surrounding melanocytes after UV exposure.^49^ NO production by UV-stimulated keratinocytes stimulates melanogenesis by affecting melanocyte proliferation and melanin synthesis.^50^ In addition, NO and α-MSH contribute to hyperpigmentation by increasing eumelanogenesis.^51^

NAD-dependent protein deacetylase sirtuin is simply referred as “sirtuin.” Sirtuins are a family of enzymes that play an important role in protein deacetylation, cell metabolism, and aging processes. Sirtuin 1 and Sirtuin 2 increase melanin synthesis by increasing the proliferation of melanocytes. Sirtuin inhibitors suppress melanin synthesis.^52^

In a study conducted by Shieh et al. (2010), it was stated that Caspase-9 causes apoptosis in melanoma cells through mechanisms such as endoplasmic reticulum stress, changes in mitochondrial membrane potential and ROS activation.^53^

Studies have shown that different types of adrenergic receptors, such as α-2 adrenergic receptors and beta (β)-adrenergic receptors, play a role in regulating pigment distribution and melanin production in various cells.^54^^,^^55^ In addition to these targets, metformin also interacts with histamine H4 receptors and L-ascorbic acid interacts with sphingosine 1-phosphate receptor 2 (S1PR2), estrogen receptors and glutamate receptors. Histamine, a biogenic amine, plays an important role in various physiological processes, including melanin synthesis. The histamine H4 receptor (H4R) is a relatively newly discovered member of the histamine receptor family. Studies have shown that histamine can induce melanogenesis in human melanocytes through protein kinase A activation.^56^

Sphingosine 1-phosphate (S1P) is a lipid that plays a role in many biological processes such as cell proliferation, angiogenesis, inflammation, and melanin synthesis.^57^ S1P receptors, especially the S1PR2 receptor, are involved in processes such as proliferation and differentiation of keratinocytes, which are necessary for melanin synthesis.^58^ In addition to S1P, we found that L-ascorbic acid also interacts with estrogen and glutamate receptors. When estrogen binds to its receptors on melanocytes and keratinocytes, it induces the synthesis of proteins such as tyrosinase, TRP-1, TRP-2 and MITF, resulting in melanin production.^59^ Furthermore, according to Hoogduijn et al. (2006), melanocytes also express the ionotropic glutamate receptors GluR2, GluR4, and N-methyl-D-aspartate (NMDA) receptors (NMDAR).^60^ The increase in melanin synthesis and TYR activation seem to be related to the NMDAR in different species.^3^

## Conclusion

In conclusion, various studies are currently being conducted to develop new therapeutic agents to treat melasma. Studies show that some drugs already in use (i.e. metformin and ascorbic acid) can be used in different areas with “drug repositioning” and such studies have gained momentum in recent yr. There is not much data in the literature about the use of metformin as a new treatment option for the treatment of melasma. When our results with MNT-1 cells are evaluated in a common framework, we can suggest that metformin may be a better treatment option than ascorbic acid. However, their co-administration may not provide an advantage. More detailed in vitro studies should be performed on this subject. The results obtained from in vitro studies should be confirmed with animal experiments and clinical trials. This may provide more realistic results on whether metformin may be a suitable treatment option for hyperpigmentation or not.
